# Friction and Heat Transfer in Membrane Distillation Channels: An Experimental Study on Conventional and Novel Spacers

**DOI:** 10.3390/membranes12111029

**Published:** 2022-10-22

**Authors:** Nunzio Cancilla, Alessandro Tamburini, Antonino Tarantino, Salvatore Visconti, Michele Ciofalo

**Affiliations:** Dipartimento di Ingegneria, Università di Palermo, Viale delle Scienze Ed. 6, 90128 Palermo, Italy

**Keywords:** membrane distillation, heat transfer, pressure drop, spacer-filled channel, spheres spacer, thermochromic liquid crystals

## Abstract

The results of an experimental investigation on pressure drop and heat transfer in spacer-filled plane channels, which are representative of Membrane Distillation units, are presented and discussed. Local and mean heat transfer coefficients were obtained by using Thermochromic Liquid Crystals and Digital Image Processing. The performances of a novel spacer geometry, consisting of spheres that are connected by cylindrical rods, and are hereafter named spheres spacers, were compared with those of more conventional woven and overlapped spacers at equal values of the Reynolds number Re (in the range ~150 to ~2500), the pitch-to-channel height ratio, the flow attack angle and the thermal boundary conditions (two-side heat transfer). For any flow rate, the novel spacer geometry provided the least friction coefficient and a mean Nusselt number intermediate between those of the overlapped and the woven spacers. For any pressure drop and for any pumping power, the novel spacer provided the highest mean Nusselt number over the whole Reynolds number range that was investigated. The influence of buoyancy was also assessed for the case of the horizontal channels. Under the experimental conditions (channel height *H* ≈ 1 cm, Δ*T* ≈ 10 °C), it was found to be large in empty (spacer-less) channels that were up to Re ≈ 1200 (corresponding to a Richardson number Ri of ~0.1), but it was much smaller and limited to the range Re < ~500 (Ri < ~0.5) in the spacer-filled channels.

## 1. Introduction

Membrane distillation (MD) is a separation process in which two currents at different temperatures, called the feed and the permeate, are separated by an interposed hydrophobic membrane [1,2,3]. The driving force for the separation is the trans-membrane vapor pressure difference, which is caused in its turn by the temperature difference between the feed (hot fluid) and the permeate (cold fluid). The membrane rejects the liquid and the non-volatile components while allowing the passage of vapor, which condenses on the permeate side. Membranes in sheets are used in the plate-and-frame or spiral-wound modules.

Several MD configurations exist, differing in the method that is used to process the permeate. The relative advantages and disadvantages of the various configurations have been discussed, for example, in a recent review study by Gurreri et al. [3]. Here, only a brief synthesis of that review is reported.

In direct contact membrane distillation (DCMD), the membrane is in direct contact with both of the liquid phases, and the permeate condenses in a cold liquid stream. The DCMD configuration allows high permeate fluxes, but it exhibits a large amount of heat loss by conduction. In air-gap membrane distillation (AGMD), the permeate condenses on a condensation surface, which is separated from the membrane by a stagnant air layer and cooled by a cold stream flowing in a third channel. This configuration reduces the conductive heat losses and allows for an internal heat recovery by pre-heating the cold feed, thus increasing the energy efficiency, but it introduces an additional resistance to mass transfer, which reduces the flux. In vacuum membrane distillation (VMD), the water vapor is extracted by a vacuum pump and condenses outside the module. VMD is characterized by a low amount of heat loss by conduction, but also by a higher risk of pore wetting (see below). In sweeping gas membrane distillation (SGMD), a gas is used as a carrier for the water vapor, which then condenses outside the MD module. The amount of heat loss by conduction is reduced and the mass transfer, when it is compared to AGMD, is enhanced by the gas flow. DCMD and AGMD are suitable for the desalination and concentration of aqueous solutions, while VMD and SGMD are suitable for the removal of volatile compounds (e.g., organics). Other designs have also been developed, including permeate gap, material gap, and vacuum multi-effect MD.

Whatever the specific design that is adopted, an important factor eventually limiting mass transfer in membrane distillation is the thermal resistance that is associated with convective heat transfer from the feed bulk to the membrane surface (feed-side temperature polarization), while the condensate-side thermal resistance is usually much smaller [4]. Polarization is commonly contrasted by placing in the feed-channels spacers in an apt location to promote mixing, thus enhancing the convective heat transfer coefficient [5].

Unfortunately, the spacers also cause a significant increase in the friction coefficient, thus enhancing pressure drop and pumping power. While this latter item is usually negligible in terms of the cost when it is compared to the other main cost components of MD, the former issue is more critical since it is associated with high trans-membrane pressures which may exceed the so-called Liquid Entry Pressure (LEP), beyond which the hydrophobic membrane is wetted, and its selectivity drops drastically. For most commercial membranes, the LEP varies between one and a few bars [6].

Therefore, most of the ongoing research on better spacer designs is focused on the double purpose of increasing the fluid mixing, and thus, the heat transfer coefficients, while keeping the frictional pressure losses within the acceptable values [7,8,9,10,11]. A comparison of two of the most popular spacer designs, i.e., the overlapped and woven ones, is given by Gurreri et al. [12] for a pitch-to-height ratio *P*/*H* = 2. The results showed that the woven spacers provided higher Nusselt numbers, but there were also higher friction coefficients with respect to the overlapped spacers. When the two parameters were considered together, the woven spacers were found to yield higher values of the mean Nusselt number for any pumping power consumption.

A novel spacer geometry for mixing promotion in the general membrane processes was proposed and computationally investigated by Koutsou and Karabelas [13] and later by Chong et al. [14]. It consists of spheres that are connected by cylindrical rods so that the membrane-spacer contact is limited to isolated spots. This geometry does not seem to have been since studied experimentally. The main motivation of the present work is to characterize the behavior of this spacer design in terms both of the pressure drop and of the heat transfer enhancement and to compare it with more traditional geometries (overlapped and woven) which have been thoroughly investigated in recent years by the authors [15,16].

## 2. Materials and Methods

### 2.1. Spacers

**Figure 1:**
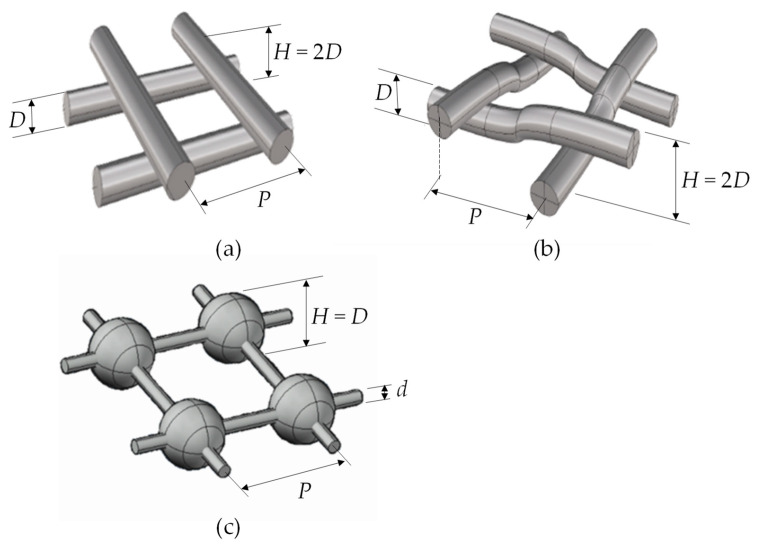
Schematic representation of the three geometries that were tested with main nomenclature: *H* = channel thickness, *D* = sphere diameter, *P* = pitch, *d* = diameter of the connecting rods. (**a**) overlapped; (**b**) woven; (**c**) spheres.

Figure 1 shows the three spacer geometries that were tested and the relevant nomenclature. The spacers were purpose assembled from PVC rods (overlapped), rubber wire (woven) and plastic spheres/sticks (spheres), and they were scaled ~3:1 with respect to the common MD spacers; this allowed for a better control of the geometry and a better compliance with the assumption of one-dimensional heat flow (see Section 2.3).

In all of the cases, the spacer filaments were arranged in two arrays at 90° with respect to each other; *H* denotes the channel thickness and *P* the pitch (distance between consecutive filaments). In all of the tests that were performed, the pitch-to-height ratio was *P*/*H* = 4 and the spacers were lying in a horizontal plane, i.e., orthogonally to the gravity acceleration vector. For the woven and overlapped geometries, the dimensions were *H* = 1 cm and *P* = 4 cm. For the spheres’ geometry, for availability reasons, the sphere diameter (equal to the channel thickness *H*) was 1.1 cm and the pitch *P* was 4.4 cm, while the diameter *d* of the connecting rods, or filaments, was 2 mm. Both in the spheres and in the woven geometry, the contact between the spacer and each membrane occurred at isolated points, while in the overlapped geometry it occurred along the whole lines, which was expected to cause a larger “spacer shadow” effect. In the spheres spacer, the connecting filaments were “floating” in the fluid.

Two spacer orientations with respect to the flow direction were tested: 0–90° (flow direction forming attack angles *ϕ* of 0° with one array of filaments and 90° with the other) and 45° (flow direction bisecting the right angle formed by the two filament arrays). In the overlapped spacers with *ϕ* = 0–90°, if the buoyancy effects are neglected, the resulting wall heat transfer coefficient *h* is expected to differ markedly both in the distribution and in the mean value between the two opposite walls. In all of the other configurations, for symmetry reasons, the two distributions were expected to be the same (discounting reflections), and to exhibit the same mean value.

It was supposed that when also buoyancy effects are taken into account, a further difference in the heat transfer coefficient (both in distribution and in mean value) will occur between the top and the bottom walls, independent of the spacer’s type and orientation (see Section 3.2).

**Figure 2:**
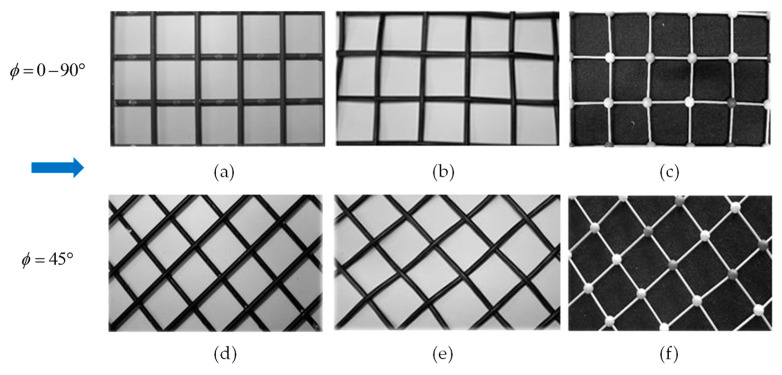
Photographs of the actual spacers tested, with their filaments oriented at 0–90° (**top** row) or at 45° (**bottom** row) with respect to the flow direction (arrow). (**a**,**d**) overlapped; (**b**,**e**) woven; (**c**,**f**) spheres. The pitch-to-height ratio *P*/*H* = 4 in all of the cases.

Figure 2 reports the real photographs of the three spacer geometries that were tested in both of the possible orientations with respect to the flow direction.

### 2.2. Test Section

**Figure 3:**
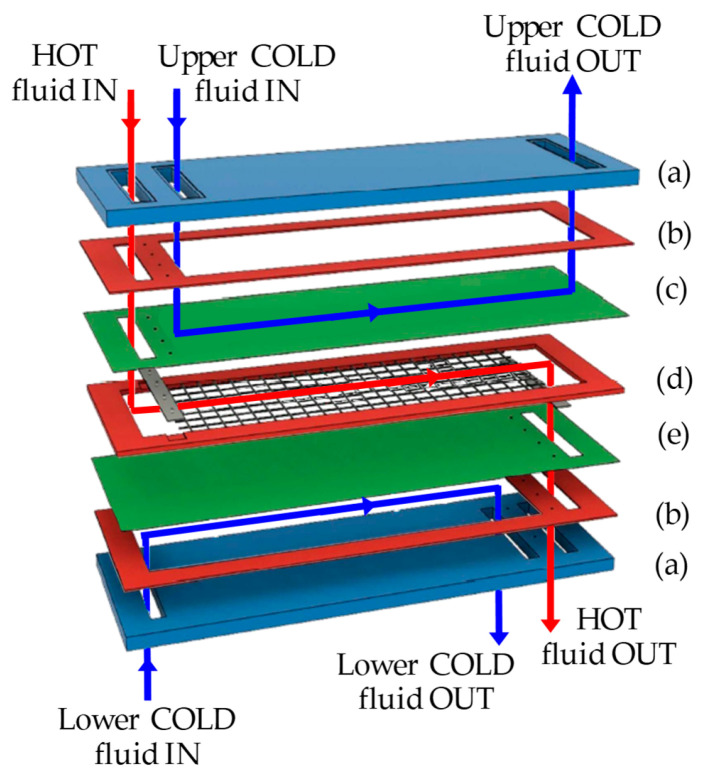
Exploded view of the test section, showing an overlapped spacer. (a) Transparent Plexiglas^®^ slab; (b) rubber gasket; (c) upper PC/TLC sandwich; (d) gasket and spacer; (e) lower PC/TLC sandwich.

The test section is schematically shown in Figure 3, and it is described in detail in Ciofalo et al. [17]. The working fluid is distilled water. The test section includes a spacer-filled hot channel and two symmetric empty cold channels to allow for two-side heat transfer. With respect to a real MD unit, on either side of the hot channel, the membrane is replaced by an impermeable wall that is cooled from the opposite side. The rationale for this is that convection heat transfer from the bulk of the hot fluid (feed) to the wall is independent of the nature of the wall itself. In a MD module, the heat that is exchanged with each membrane crosses this latter partly by conduction (as sensible heat) and partly in the form of a vapor flux (as latent heat), while in the present test section, this second contribution is absent.

Between the hot channel and either of the cold channels, a sandwich is interposed, consisting of a Thermochromic Liquid Crystal (TLC) foil that was placed between two identical polycarbonate (PC) sheets, which are 1 mm each in thickness. The TLC foils (commercialized by Edmund Optics^®^_,_ Barrington, IL, USA) were characterized by a Red Start at 25 °C and a Colour Play of 5 °C (blue start at 30 °C).

The thickness of both of the cold channels is *H_c_* = 4 mm, while the thickness of the hot channel is defined by the spacer thickness, and thus, it is *H_h_* = 10 mm in the presence of the overlapped or woven spacers and *H_h_* = 11 mm in the presence of the spheres spacer.

The test section is inserted in a rig that is provided with two centrifugal circulation pumps, a Corema Junior^®^ chiller (Riley Surface World, Walsall, UK) for the coarse regulation of the cold fluid temperature, a Julabo^®^ (Seelbach, Germany) cooling thermostatic bath for the fine adjustment of the same temperature, and a second Julabo^®^ thermostatic bath for the regulation of the hot fluid temperature. The instrumentation includes a Fuji Electric^®^ FCX-AII differential pressure transmitter (Tokyo, Japan), six Pt100 thermoresistance probes and three Krohne Optiflux^®^ (Duisburg, Germany) magnetic flow meters (one for the hot channel and one for each of the two cold channels, respectively).

### 2.3. Experimental Technique

**Figure 4:**
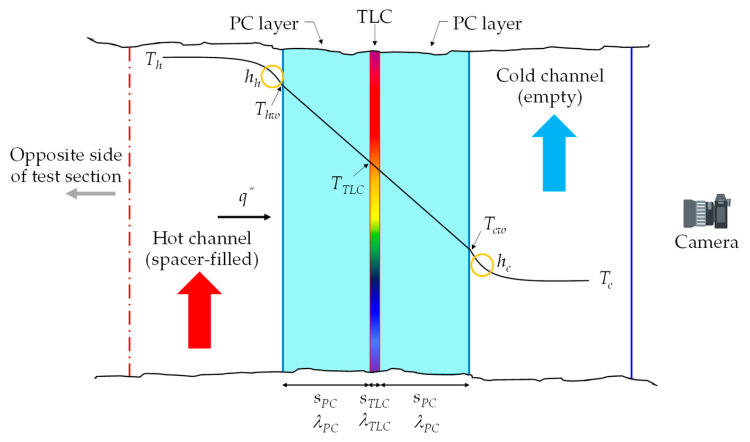
Schematic of heat transfer across the PC-TLC sandwich located on one of the two sides of the test section. *T_h_* = bulk temperature of the hot fluid, *T_c_* = bulk temperature of the cold fluid, *T_cw_* = cold wall temperature, *T_hw_* = hot wall temperature, *T_TLC_* = TLC temperature, *h_h_* = convective heat transfer coefficient on the hot side, *h_c_* = convective heat transfer coefficient on the cold side, *q^″^* = heat flux, *s_PC_* = thickness of the PC sheet, *s_TLC_* = thickness of the TLC foil, *λ_PC_* = thermal conductivity of the PC sheet, *λ_TLC_* = thermal conductivity of the TLC foil.

Figure 4 schematically shows the process of heat transfer across one of the two PC-TLC sandwiches.

On each side of the test section, the active surface of the relevant TLC sheet was photographed using a digital camera through the ~0.2 mm-thick front polyester cover of the TLC sheet itself; one of the two 1 mm-thick PC sheets made up the relevant sandwich; the 4 mm-thick cold channel; the 30 mm-thick Plexiglas^®^ slab which constitutes the external wall of the test section. The images were then digitally processed as described in detail in [17] to obtain, based on a previous in situ calibration, the 2-D distribution of the TLC temperature, *T_TLC_*. Only a central portion of the TLC sheet including an integer number of unit cells of the spacer lattice was used for the analysis. At each point, the bulk temperatures *T_h_* and *T_c_* of the hot and cold channels were obtained as functions of the coordinate *x* along the flow direction by interpolating between the readings of the Pt100 probes that were located near the inlets and outlets of each channel.

Under a one-dimensional heat transfer assumption (heat flowing orthogonally to the layers), by imposing the heat flux from *T_h_* to *T_TLC_* to be equal to that from *T_TLC_* to *T_c_* and using the elementary formulae for thermal resistances in a series, the following expression was obtained for *h_h_*:

(1) $$h_{h}=\frac{\left(T_{TLC}-T_{c}\right)}{T_{h}\left(\frac{s_{PC}}{\lambda_{PC}}+\frac{s_{TLC}}{\lambda_{TLC}}+\frac{1}{h_{c}}\right)-T_{TLC}\left(2\frac{s_{PC}}{\lambda_{PC}}+\frac{s_{TLC}}{\lambda_{TLC}}+\frac{1}{h_{c}}\right)+T_{c}\left(\frac{s_{PC}}{\lambda_{PC}}\right)}$$

in which *λ* is the thermal conductivity and the other symbols are defined in Figure 4; the subscripts *PC* and *TLC* represent the polycarbonate and Thermochromic Liquid Crystals, while the subscripts *h* and *c* represent the hot and cold fluids, respectively.

Under the same 1-D assumption, the following expressions were applied for the heat flux *q*″ and the hot wall temperature *T_hw_*:

(2) $$q\prime \prime =\frac{T_{TLC}-T_{c}}{\frac{s_{PC}}{\lambda_{PC}}+\frac{s_{TLC}}{\lambda_{TLC}}+\frac{1}{h_{c}}}$$

(3) $$T_{hw}=\frac{T_{TLC}\left(2\frac{s_{PC}}{\lambda_{PC}}+\frac{s_{TLC}}{\lambda_{TLC}}+\frac{1}{h_{c}}\right)-T_{c}\left(\frac{s_{PC}}{\lambda_{PC}}\right)}{\left(\frac{s_{PC}}{\lambda_{PC}}+\frac{s_{TLC}}{\lambda_{TLC}}+\frac{1}{h_{c}}\right)}$$

Identical formulae were used in [17] for the same layout of the test section.

Actually, the problem of deriving the 2-D surface distribution of *h_h_*, *q*″ and *T_hw_* from the 2-D distribution of *T_TLC_* over a plane that was embedded in a solid (PC-TLC “sandwich”) is a steady-state inverse heat conduction problem, and it can be given more accurate, but also much more complex, solutions by the use of various inverse heat conduction methods, one of which is specifically adapted to the present problem (third type thermal boundary conditions on one wall), and it is based on the 3-D solutions of the corresponding direct conduction problems, and it has recently been presented by one of the present authors [18].

However, under the present experimental conditions (a relatively thin PC-TLC “sandwich”) the difference in the results between the complete 3-D approach and the simple 1-D approach used in the present paper is small and would not justify the (much greater) computational effort that is required by the 3-D method, especially in view of the large number of tests that were performed. Ideally, the assumption of 1-D heat transfer in the PC-TLC sandwich would be exactly satisfied for a slab that is infinitely thin with respect to the channels; the adoption of the scaled-up spacers was chosen also in order to satisfy this condition more closely.

An independent assessment of the adequacy of the one-dimensional heat transfer assumption for a similar problem was also presented in another paper by our research group [19], and we arrived at similar conclusions.

The local heat transfer coefficient *h_h_* can be expressed in a dimensionless form as a local Nusselt number

(4) $${\mathrm{Nu}}_{h}=h_{h}\cdot \frac{D_{eq}}{\lambda_{h}}$$

where *D_eq_* is the hydraulic diameter of the channel, which is conventionally identified with 2*H_h_* as in a spanwise indefinite channel, and *λ_h_* is the hot fluid thermal conductivity, which is a weak function of its bulk temperature *T_h_*.

The mean Nusselt number (relative to either wall) is defined here as

(5) $$\mathrm{Nu}=\frac{\langle q\prime \prime \rangle }{\lambda_{h}\left(\langle T_{hw}\rangle -\bar{T_{h}}\right)}$$

where the brackets 〈·〉 denote surface averages over the portion of interest of the wall, corresponding to the TLC area that was used for the analysis, while an overbar denotes the corresponding average along the flow direction. The above definition was preferred to the mere surface average 〈Nu*_h_*〉 of the local Nusselt number since this latter quantity may become singular at the points where the denominator in Equation (1) vanishes.

For the estimate of the cold-side heat transfer coefficient *h_c_*, necessary to compute *h_h_* from Equation (1); for the assessment of entry effects and for a sensitivity analysis including the estimate of the measurement uncertainty, we direct the reader to a previous paper [17] in which these issues are thoroughly discussed.

## 3. Results and Discussion

In the present work, the Reynolds number (Re) is defined as:

(6) $$\mathrm{Nu}=\frac{\langle q\prime \prime \rangle }{\lambda_{h}\left(\langle T_{hw}\rangle -\bar{T_{h}}\right)}$$

where *D_eq_* = 2*H_h_* as in Equation (4), U is the mean velocity of the fluid and *ρ* and *µ* are its density and dynamic viscosity, respectively.

The aim of the present experimental study was to characterize the heat transfer in the spacer-filled channels under conditions that were representative of membrane distillation, with Reynolds numbers that were in the range from laminar to transitional and early turbulent (~150–2500). In the presence of the spacers, the transition to the unsteady and turbulent flow (as indicated by the time-dependence of the TLC colour patterns in previous studies, e.g., [15]) occurred at a Reynolds number of ~400–500.

### 3.1. Friction Coefficient

The Darcy friction coefficient *f* is defined as:

(7) $$f=\frac{2\Delta pD_{eq}}{L\rho U^{2}}$$

in which *L* is the distance between the two pressure tappings (~0.5 m in the present tests) and Δ*p* is the measured pressure drop between them.

**Figure 5:**
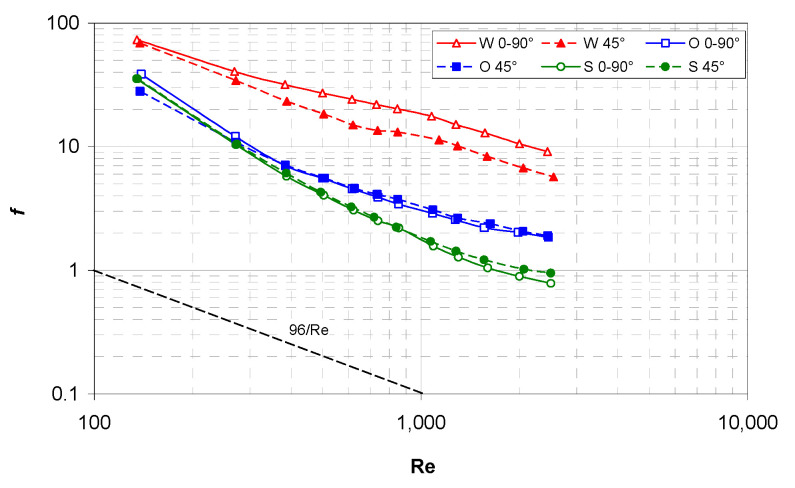
Darcy friction coefficient *f* as a function of the Reynolds number for all of the configurations investigated. The theoretical friction coefficient for laminar flow in a plane channel (96/Re) is also reported for comparison purposes.

Figure 5 reports the measured Darcy friction coefficient *f* as a function of the Reynolds number Re for all six combinations of spacer geometry (woven, overlapped and spheres) and orientation (0–90° or 45°).

The results show that the flow attack angle affected *f* significantly only in the case of the woven spacers, where the 0–90° orientation yielded values of *f* that are up to 60% higher than they are in the 45° orientation. For the other two geometries (overlapped and spheres spacers), the difference is much lower (~10% or less).

By far the highest values of *f* are associated with the woven spacer, while the lowest values are provided by the spheres spacer. The advantage of this latter result with respect to conventional spacers increases with Re; at the highest Re that was tested (2500), the *f* (spheres) is about one half of *f* (overlapped) and about one tenth of *f* (woven).

For comparison purposes, Figure 5 reports also the Hagen-Poiseuille behavior of *f* for the laminar flow in an indefinite plane channel (96/Re); it can be observed that the friction increase caused by the spacers ranges that are between ~1.5 orders of magnitude (spheres) and ~2 orders of magnitude (woven).

### 3.2. Influence of Buoyancy

Preliminarily, in order to assess the possible effects of thermal buoyancy, the measurements of the heat transfer coefficient were performed with no spacer in the hot channel using the same TLC-based technique as that which was used for the spacer-filled channels (see Section 2.3). Note that for laminar parallel flow in a plane channel with the two-side cooling, the Nusselt number varies depending on the exact thermal boundary conditions, between ~7.54 (uniform *T_hw_*) and ~8.24 (uniform *q*″) [20].

**Figure 6:**
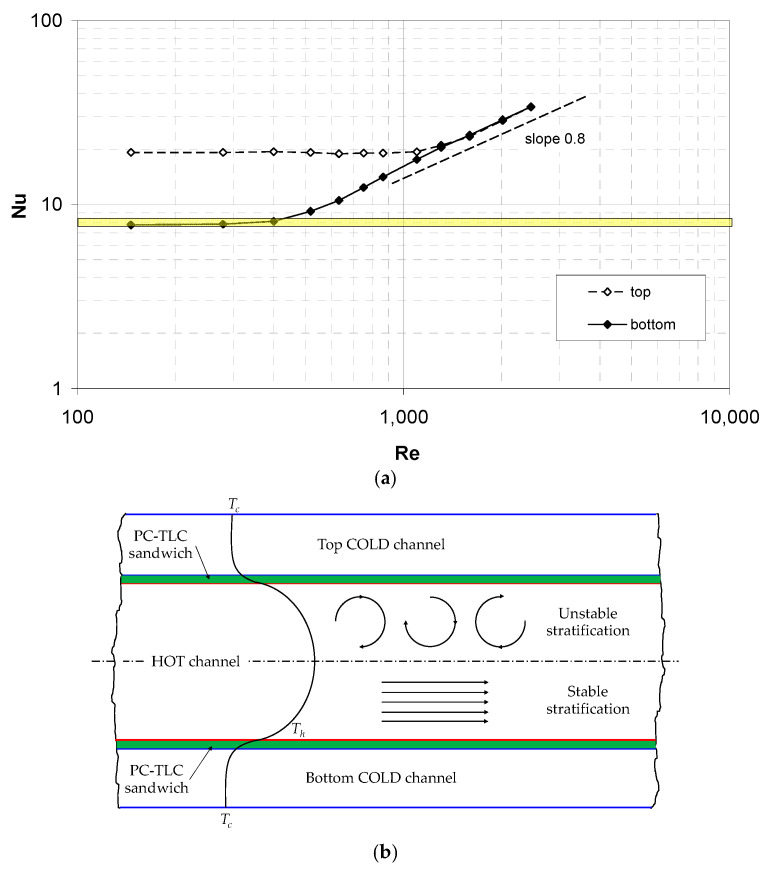
(**a**) Experimental mean Nusselt number as a function of the Reynolds number on the top and bottom walls of the test section in the case of no spacer, showing the influence of buoyancy. The shaded area represents the expected range of Nu in the absence of buoyancy (7.54–8.34). (**b**) Schematic representation of the temperature distribution in the hot channel, with unstable stratification in its upper half and stable stratification in its lower half.

Figure 6a reports the mean Nusselt number, Equation (5), on the top and bottom walls. The experimental data exhibit a behaviour that is completely different from the above theoretical predictions. The Nusselt number on the bottom wall remains constant at a value of ~7.8, which is intermediate between the two above theoretical limits, only up to Re ≈ 300–400 and then increases strongly. On the other hand, the Nusselt number on the top wall remains approximately constant at a much larger value (~19) up to Re ≈ 1200. For larger values of Re, the two Nusselt numbers merge and increase with the Re following a ~0.8-power law, which is characteristic of the turbulent flow in the simple channels.

This strongly asymmetric behaviour can only be explained by the influence of thermal buoyancy on the basis of the schematic representation of the temperature distribution in the hot channel in Figure 6b. In the upper half of the channel, an unstable thermal stratification exists, which causes convective motions and a strong heat transfer enhancement even at very low Re. On the other hand, in the lower half of the channel, the thermal stratification is stable, causing the flow to remain laminar and parallel and the Nusselt number to settle to a value that is compatible with the theoretical parallel-flow predictions. 

The above qualitative remarks can be made quantitative on the basis of the definitions of the Rayleigh (Ra) and Richardson (Ri) numbers:

(8) $$\mathrm{Ra}=\frac{g\beta \left(T_{h}-T_{hw}\right){\left(H_{h}/2\right)}^{3}}{\nu \alpha }$$

(9) Ri=RaRe2

in which *g* is the acceleration due to gravity, *β*, *ν* and *α* are the thermal expansion coefficient, the kinematic viscosity and the thermal diffusivity of the fluid, respectively, while the channel height *H_h_*, the hot wall temperature *T_hw_* and the hot fluid bulk temperature *T_h_* have been defined earlier. The Rayleigh number was computed on the basis of the channel half-height, *H_h_*/2.

Under typical test conditions, one has *T_h_* ≈ 38 °C and *T_hw_* ≈ 35.5 °C, while *H_h_* = 1.1·10^−2^ m, *g* = 9.81 m/s^2^ and, for water at ~38 °C, *β* = 3.21·10^−3^ K^−1^, *ν* = 6.65·10^−7^ m^2^/s and *α* = 1.56·10^−7^ m^2^/s, thereby yielding Ra = 1.26·10^5^. Therefore, the Richardson number, which measures the importance of the natural convection with respect to the forced convection, is (for example) ~3.16 for Re = 200, ~0.35 for Re = 600 and ~0.039 for Re = 1800. These figures explain why the influence of natural convection (thermal buoyancy) is negligible at the high Reynolds numbers, important at the intermediate Re (e.g., 600) and dominating at a very low Re.

### 3.3. Mean Nusselt Number in Spacer-Filled Channels

**Figure 7:**
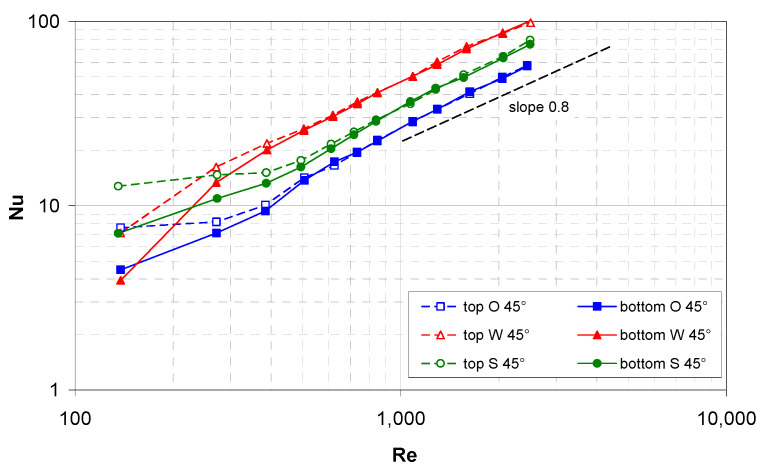
Experimental mean Nusselt number on the top and bottom walls as a function of the Reynolds number for a flow attack angle of 45° and the three spacer geometries investigated.

Figure 7 reports the mean Nusselt number on the top and bottom walls of the hot channel for the three spacers (O = overlapped, W = woven and S = spheres), whereby all of them are arranged at *ϕ* = 45° with respect to the flow direction. In these configurations, for symmetry reasons, any difference between the Nusselt numbers on the two walls is only due to buoyancy effects (apart from experimental uncertainties).

In the presence of the spacers, the effects of buoyancy (promoting heat transfer in the top half of the channel) extended only up to Re ≈ 500 (Richardson number Ri ≈ 0.5), and these effects are much smaller than they are in the spacer-less channel. A possible reason is that the spacer hinders the formation of the buoyancy-induced secondary motions that are responsible for heat transfer enhancement, while they cause the buoyancy-independent mixing. For Re>~500, the Nu curves pertaining to the two walls merge and increase in all of the cases following a ~0.8-power law as is typical of turbulent flow in simple channels. The highest values of Nu are provided by the W spacer, the lowest ones are provided by the O spacer and intermediate values are provided by the novel S spacer.

**Figure 8:**
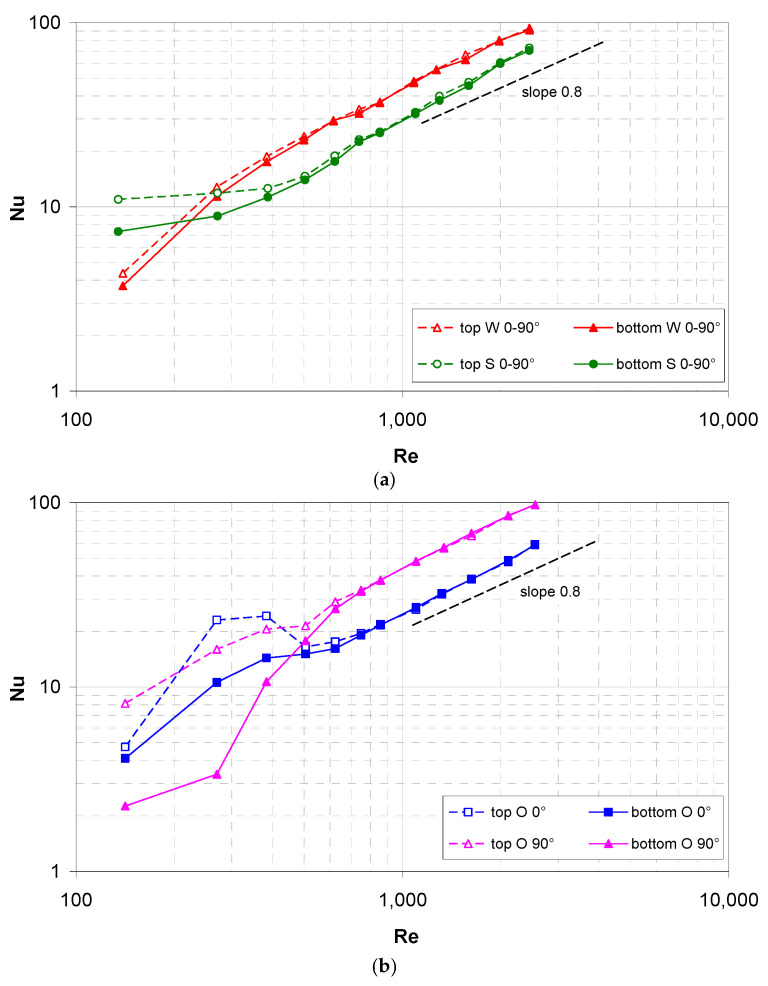
Experimental mean Nusselt number as a function of the Reynolds number on the top and bottom walls for a flow attack angle of 0° or 90°. (**a**) Woven and spheres geometries; (**b**) overlapped geometry.

Figure 8 reports similar results for *ϕ* = 0–90° (spacer filaments parallel and orthogonal to the flow direction). For clarity purposes, the results for the W and S spacers on one hand, and for the O spacer on the other hand are shown in separate graphs.

In particular, graph (a) pertains to the woven (W) and spheres (S) spacers that are arranged at 0–90°. For symmetry reasons, also in this orientation the W and S spacers are expected to exhibit the same mean Nusselt numbers on the two walls apart from the effects of buoyancy. The results are much similar to those that are reported in Figure 7 for the 45° orientation: the influence of buoyancy extends only up to Re ≈ 500 (Ri ≈ 0.5) and it is small, especially for the W spacer. As in the 45° orientation, the woven spacer yields values of Nu that are higher than they are for the spheres spacer.

Graph (b) pertains to the overlapped (O) spacers that were arranged at 0–90°. In this case, even in the absence of buoyancy, different mean Nusselt numbers were expected on the two opposite walls, according to whether the wall-adjacent spacer filaments were arranged at 0° or 90° with respect to the flow direction. The presence of buoyancy further changes the results according to whether a given wall is placed on the top or on the bottom of the channel, and so four different curves are to be considered.

Unlike the previous results for the W and S spacers, the present ones exhibit a large and complex influence of buoyancy, suggesting that the overlapped spacers interfere less with the buoyancy-induced motions. A maximum in Nu at Re ≈ 300–400 can even be observed on the 0° side when this is placed at the top. However, also in the present case, the effects of buoyancy become insignificant in the range Re > ~500, wherein the top and bottom curves for each orientation merge and follow the ~0.8-power law. In this range, the mean Nusselt number is almost double on the 90° wall (where the wall-adjacent spacer filaments are orthogonal to the flow) that on the 0° wall (where the wall-adjacent spacer filaments are parallel to the flow). In comparison with the woven and spheres geometries, Nu is slightly larger than with the W spacers on the 90° wall, and it is smaller than the S spacers on the 0° wall.

The strong asymmetry between the 0°-wall and the 90°-wall in terms of heat transfer makes the O 0–90° configuration unsuitable for MD applications, where, for thermodynamic reasons, it is desirable to achieve the same overall heat (and thus, water vapor) transfer from the two sides of the hot (feed) channel.

### 3.4. Performance Comparison

In the context of membrane distillation, the main performance parameters of a spacer are the mean Nusselt number Nu and the friction coefficient *f*. However, they have a rather different impact on the design and cost of a MD unit: for any given product (freshwater) outflow, a high mean Nusselt number allows a reduction in the membrane surface and/or of the driving temperature difference, with direct benefits on the plant’s cost and/or on the cost of the required thermal energy, which are the most relevant cost items. On the other hand, the benefits of a low friction coefficient are usually small in terms of the pumping power savings, but they may become crucial in terms of the design if LEP issues are involved.

Therefore, comparisons being made between the alternative spacers based on a single figure of merit (e.g., Nu/*f*) are inappropriate. A better comparison method is based on introducing two dimensionless numbers N_Δ*p*_ = *f*·Re^2^ (pressure number, which is proportional to the inlet-to-outlet pressure drop) and N*_W_*= *f*·Re^3^ (power number, which is proportional to the required pumping power) and reporting the mean Nusselt number as a function of either parameter [21]. If the Nu(N_Δ*p*_) curve for spacer A lies above that for spacer B, then spacer A will provide a better performance for any pressure drop, while, if the Nu(N_W_) curve for spacer A lies above that for spacer B, then spacer A will provide a better performance for any pumping power consumption.

**Figure 9:**
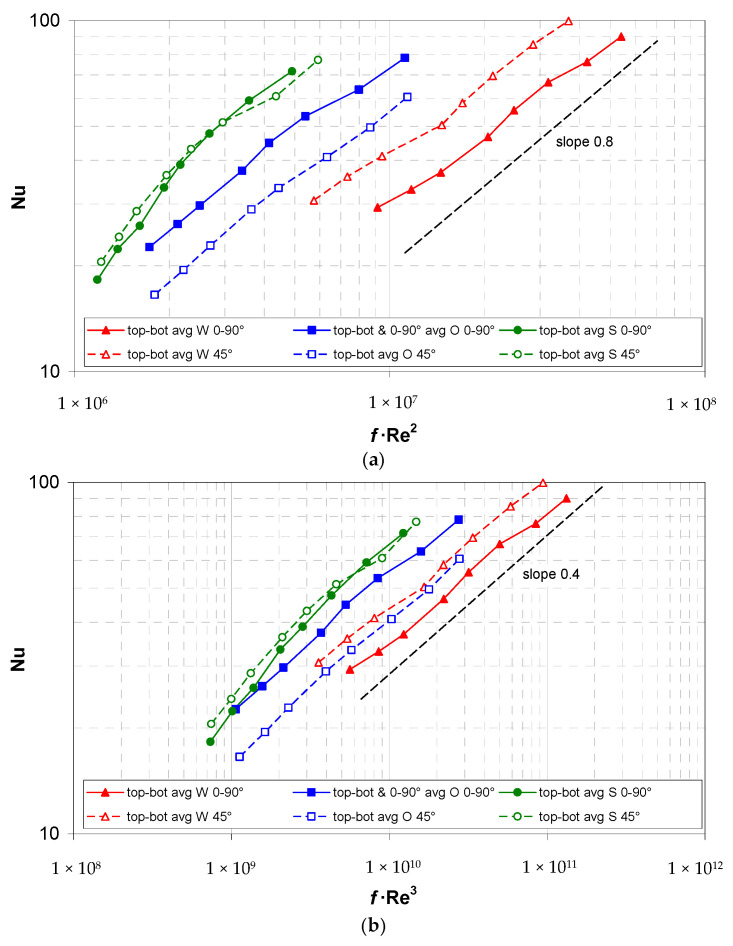
Experimental mean Nusselt number (top-bottom averaged) as a function of the pressure number *f*·Re^2^ (**a**) or the power number *f*·Re^3^ (**b**) for all the spacer configurations investigated.

For all of the spacer configurations that were investigated, both of the above types of curves are shown in Figure 9a,b. For the sake of clarity, in all of the cases, the reported mean Nusselt number is an average between the top and bottom values, and only cases with Re > 500, wherein it was negligibly affected by the buoyancy effects, are included. Moreover, in order to allow for an easy comparison with the W and S geometries, for the overlapped (O) spacer that was oriented at 0–90° with respect to the flow direction also the relevant 0° and 90° values have been averaged.

The graphs show that, among the geometries that were tested, in the range Re > 500 and regardless of the spacer orientation, the best performance was provided by the novel spheres geometry both for any given pressure drop and for any given pumping power, whereas the worst performance was provided by the woven geometry at 0–90°, despite its high Nu values. Note that, at the present pitch-to-channel height ratio *P*/*H* = 4, the ranking of the overlapped and woven spacers is different from that which was observed in the comparative study [12], which was for *P*/*H* = 2. Note also that thanks to the favourable friction coefficient of the spheres spacer, the relative ranking of the spheres and the woven spacers in both of the graphs of Figure 9 is reversed with respect to those which is reported in Figure 7 and Figure 8a which have Re as the abscissa.

These results suggest that the woven spacers should be used only if an increase in the mean heat transfer coefficient, and thus a reduction in the membrane surface area, is the only or the main goal, e.g., if the membrane cost is the dominant cost item. In all of the other circumstances, the spheres spacers should be preferred both to reduce the maximum trans-membrane pressure, thereby avoiding LEP issues, and to reduce the pumping power consumption.

### 3.5. Local Heat Transfer Coefficient Distributions

The following figures report examples of the local Nusselt number distributions on the channel walls in a unit cell of the spacer lattice, which were obtained with different spacers. In all of the cases, the Reynolds number was ~500 so that buoyancy effects were negligible, and the distinction between top and bottom sides of the channel is irrelevant. All of the maps are represented from the same viewpoint; the insets below each map show the arrangement of the spacer with respect to the flow direction. In the case of the overlapped spacers, the filaments touching the wall that is under consideration are represented in a darker shade of grey.

**Figure 10:**
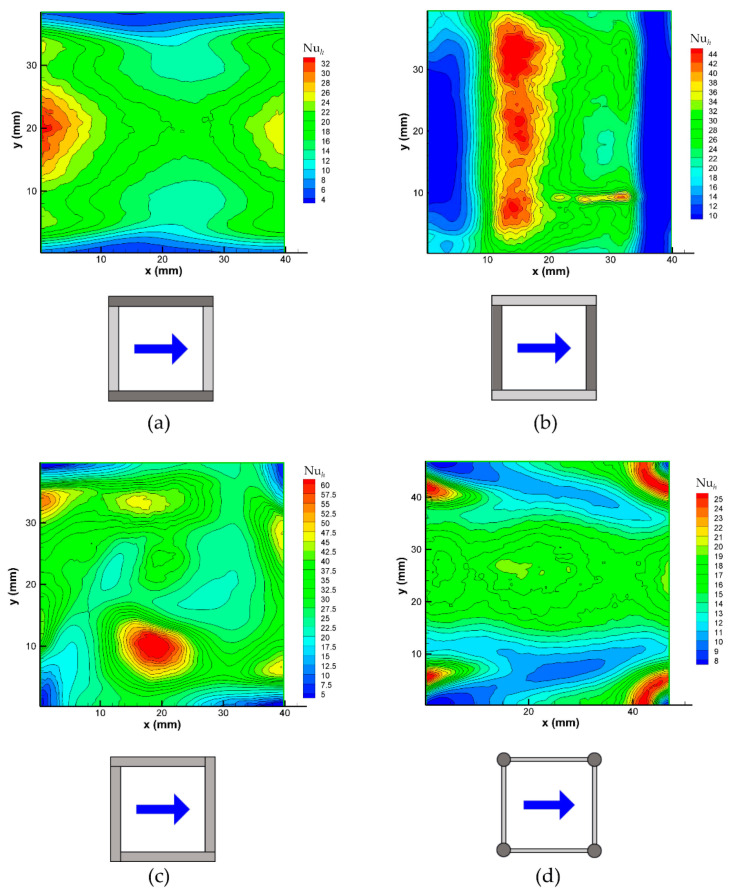
Distributions of the local Nusselt number on a wall for different spacer configurations at a Reynolds number of ~500. (**a**) Overlapped spacer, flow attack angle *ϕ* = 0°; (**b**) overlapped spacer, *ϕ* = 90°; (**c**) woven spacer, *ϕ* = 0–90°; (**d**) spheres spacer, *ϕ* = 0–90°. Note that for the woven and spheres geometries, there is no difference between the 0° and 90° attack angles.

Figure 10 shows the 0–90° orientation. 

Graph (a) pertains to the 0° wall of a channel that is filled with an overlapped spacer that is oriented at 0–90° (O 0–90°). It shows the minima of Nu*_h_* around the wall-filament contact lines, while the maxima of Nu*_h_* are attained in the central region of the sides, lying above the opposite filaments (adjacent to the 90° wall), where the flow passage area is restricted and the local velocity is at its highest. The mean Nusselt number, which was computed from Equation (5), is ~16, (see Figure 8b).

Graph (b) pertains to the 90° wall in the presence of the same spacer (O 0–90°). It shows the minima of Nu*_h_* around the wall-filament contact lines, which of course are now orthogonal to those that are represented for the opposite wall in graph (a). The maxima of Nu*_h_* are attained at about 1/3^rd^ of the pitch *P* downstream of each wall-adjacent filament in correspondence with the reattachment of the flow separating it from the upstream wall-adjacent filament. The mean Nusselt number Nu, which was computed from Equation (5), is ~19, which is only slightly larger than that on the 0° wall, as shown also in Figure 8b, while the absolute maximum of the local Nusselt number Nu*_h_* is much larger than it is on the 0° wall.

Graph (c) pertains to one wall of a channel that is filled with a woven spacer (W 0–90°). The four small contact areas at the corners of the unit cell surrounding the point-like contacts between the spacer and the wall are characterized by very low values of Nu*_h_*. As indicated by the preliminary CFD simulations, the absolute and relative maxima of Nu*_h_* correspond to the regions in which the complex flow that is induced by the spacer impacts against the wall that is under consideration. The mean Nusselt number Nu, which was computed from Equation (5), is ~23, as shown also in Figure 8a, while the absolute maximum of the local Nusselt number Nu*_h_* is ~60; both of these values are the highest among all of the configurations in this figure.

Finally, graph (d) reports the distribution of Nu*_h_* on one wall of a channel that is filled with the spheres spacer (S 0–90°). Note that the pitch *P* is now 44 mm, which is slightly larger than it is for the previous geometry. In this case, the Nu*_h_* minima at the four corners of the unit cell, which are associated with the point-like contacts between the spacer spheres and the wall, are surrounded by horseshoe structures that are characterized by high Nu*_h_* values upstream of each sphere, which are associated with the impact of the flow against this latter. Other eye-catching structures are the low Nu*_h_* wakes that result from the downstream side of each sphere. Apart from the mentioned features, the Nu*_h_* distribution over the remaining part of the wall appears to be less structured and exhibits intermediate values. The mean Nusselt number Nu, which was computed from Equation (5), is ~15, see Figure 8a, while the absolute maximum of the local Nusselt number Nu*_h_* is ~25; therefore, the spheres geometry is that which exhibits the flattest distribution of the local heat transfer coefficient. 

**Figure 11:**
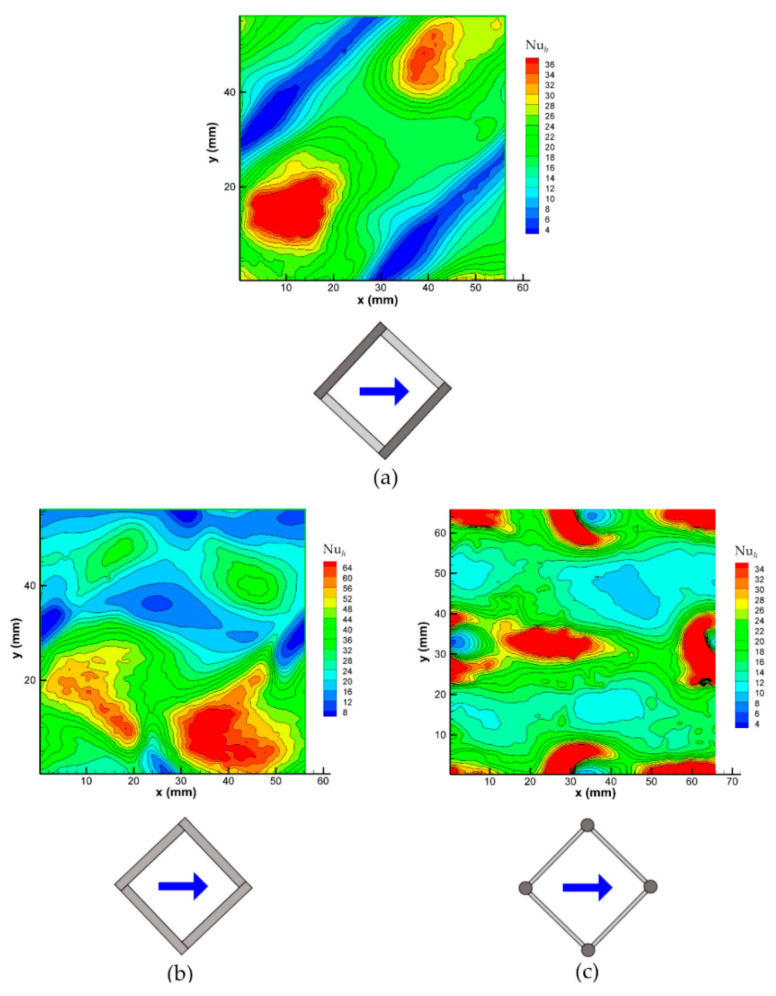
Distributions of the local Nusselt number on a wall for different spacer configurations at a Reynolds number of ~500 and a flow attack angle of 45°. (**a**) Overlapped spacer; (**b**) woven spacer; (**c**) spheres spacer.

Figure 11 shows the 45° orientation. Note that in this case, the unit cells are larger by a factor $\sqrt{2}$ (i.e., the area is double the size) with respect to those in Figure 10. 

Graph (a) pertains to one wall of a channel that is filled with an overlapped spacer (O 45°). For this orientation, due to symmetry reasons, the distributions of Nu*_h_* on the opposite walls of the hot channel are essentially identical, when we are disregarding the reflections, so it is sufficient to show a single wall. The map shows the minima of Nu*_h_* around the diagonal wall-filament contact lines, while the Nu*_h_* maxima are attained in two separate regions that are located between the filaments and presumably corresponding to the flow reattachment regions as in the O 90° case in Figure 10b. The mean Nusselt number Nu, which was computed from Equation (5), is ~14, see Figure 7, while the Nu*_h_* maxima are much higher (>36).

Graph (b) pertains to one wall of a channel that is filled with a woven spacer (W 45°). The four areas surrounding the point-like contacts between the spacer and the wall, and those that are characterized by very low values of Nu*_h_*, are now located at the centers of the four sides of the unit cell. As in the 0–90° case (Figure 10c), an isolated and intense Nu*_h_* maximum can be observed, thus corresponding as indicated by preliminary CFD simulations, to a region in which the spacer-induced flow impinges against the wall that is under consideration. The mean Nusselt number Nu, which was obtained by calculating Equation (5), is ~26, see Figure 7, while the absolute maximum of Nu*_h_* is >64; both of these values are the highest among all of the configurations in this figure.

Finally, graph (c) reports the distribution of Nu*_h_* on one wall of a channel that is filled with the spheres spacer (S 45°). As in the 0–90° orientation (Figure 10d), the Nu*_h_* minima at the four point-like sphere-wall contacts are surrounded by horseshoe structures that are characterized by high Nu*_h_* values that are upstream of each sphere. However, unlike in the 0–90° case, the wake region of each sphere exhibits a complex structure with a minimum value immediately downstream that is followed by a maximum of the same amplitude as that in the horseshoe region, which is presumably caused by the confluence of the vortex streets issuing from the 45° filaments that connect the spheres. The mean Nusselt number Nu, which was calculated using Equation (5), is ~17, see Figure 7, while the absolute maximum of Nu*_h_* is >34.

## 4. Conclusions

The pressure drop and the local and mean heat transfer coefficients were measured in the spacer-filled plane channels representative of the Membrane Distillation (MD) units. The performances of a novel spacer geometry, consisting of spheres that are connected by cylindrical rods, were compared with those of more conventional woven and overlapped spacers under the conditions of two-side heat transfer, which are closely representative of many MD applications. In all of the cases, the Reynolds numbers Re ranged between ~150 and ~2500, the pitch-to-channel height ratio *P*/*H* was equal to 4, and two flow orientations of the spacer filaments with respect to the flow direction were considered (parallel/orthogonal or inclined at 45°).

The novel (spheres) spacer geometry provided the lowest friction coefficients *f* and mean Nusselt numbers Nu that were intermediate between those of the overlapped and woven spacers when they are expressed as functions of the flow rate, which are represented in a dimensionless form by the Reynolds number Re.

When we were still using Re as the independent variable, the woven spacers provided the highest mean Nusselt numbers, but at the price of a far larger friction coefficient. The overlapped spacers provided the friction coefficients that were up to twice the value of those of the novel spheres spacers, with the difference increasing with the Reynolds number. The Nusselt number was ~30% lower than that which was provided by the novel spacer in the 45° orientation, while in the 0–90° orientation, it was ~17% lower on the wall that was adjacent to the filaments that were parallel to the flow (0° wall), but it was ~50% higher on the wall that was adjacent to the filaments that were orthogonal to the flow (90° wall). This strong asymmetry between the two sides of an overlapped spacer-filled channel in the 0–90° orientation is undesirable in MD applications.

For any pressure drop, as expressed in a dimensionless form by the pressure number *f*∙Re^2^, and for any pumping power, as expressed by the power number *f*∙Re^3^, the novel spheres spacer provided the highest mean Nusselt number over the whole Reynolds number range that was investigated.

On the basis of the above results, one may conclude that the woven spacers are preferable only if the main design objective is the increase in the mean heat transfer coefficient, and thus, the reduction in the membrane surface area (e.g., if the membrane cost is the dominant cost item or if space limitations are crucial). In all of the other circumstances, the spheres spacers are preferable to the woven and overlapped ones in order both to reduce the maximum trans-membrane pressure, primarily to avoid Liquid Entry Pressure (membrane wetting) issues, and secondarily, to reduce the pumping power consumption.

The influence of buoyancy was also assessed for the cases of the horizontal channels; under the experimental conditions, it was found to be large in the empty (spacer-less) channels up to Re ≈ 1200 (corresponding to a Richardson number Ri = Ra/Re^2^ of ~0.1), but it was much smaller and limited to the range Re < ~500 (Ri < ~0.5) in spacer-filled channels.

The present study was limited to a pitch-to-channel height ratio *P*/*H* of 4, two spacer orientations, and for the novel (spheres) geometry, a filament diameter-to-sphere diameter ratio *d*/*D* of ~0.2. It is envisaged that we should extend the study to different values of *P*/*H* and *d*/*D* and to intermediate orientations and to perform parallel CFD simulations, which are particularly desirable to obtain a picture of the flow field.

## Data Availability

Data is contained within the article.
